# T186. ASSOCIATION BETWEEN POLYMORPHISMS OF THE NEUREGULIN 1 (NRG1) GENE AND SCHIZOPHRENIA

**DOI:** 10.1093/schbul/sby016.462

**Published:** 2018-04-01

**Authors:** Junzhe Xu, Jianxin Yuan, Te-An Chen, Anna Li, Meghan Helm, Steve Dubovsky

**Affiliations:** 1 School of Medicine, SUNY at Buffalo; 2 VHAWNY Healthcare System

## Abstract

**Background:**

The dysfunction of neuregulin 1 (NRG1) is one of the plausible hypotheses for the pathogenesis of schizophrenia. The neuregulin 1 (NRG1) is located on chromosome 8p, as suggested by multiple linkage studies. The aim of this study is to clarify the contribution of polymorphisms of the neuregulin 1 (NRG1) with schizophrenia

**Methods:**

After informed consent was obtained, 100 schizophrenia patients and 100 control subjects were enrolled in this study. All subjects were administered the Diagnostic Interview for Genetic Studies (DIGS) (National Institute of Mental Health-Molecular Genetics Initiative, 1992; Nurnberger et al., 1994) by a research assistant with extensive training in this interview. Blood samples were collected in anonymously identified 10-ml Vacutainer tubes (Becton Dickinson). DNA was prepared by a modified SDS/Proteinase K procedure (Gusells et al., 1979). We genotyped polymorphism neuregulin 1 (NRG1) with the PCR-RFLP methods. The PCR products were digested by restricted enzyme.

**Results:**

We observed a significant association between the polymorphism neuregulin 1 (NRG1) and the schizophrenia (Chi-Square Test P= 0.0449).

**Discussion:**

The NRG1gene was originally identified as a susceptibility gene for schizophrenia by using a combination of a linkage and association approaches based on microsatellite markers and then using SNPs after microsatellite at risk haplotypes were identified. We found there is the frequency of the polymorphism of neuregulin 1 (NRG1) was significantly increased in schizophrenia patients. This allelic association suggests that the functional polymorphism neuregulin 1 (NRG1) may play a role in susceptibility to schizophrenia. Further study with larger sample sizes is required.

